# A five-year review of the morbidity and mortality pattern in the special care baby unit of a private-owned tertiary hospital in Nigeria

**DOI:** 10.11604/pamj.2025.50.50.41967

**Published:** 2025-02-12

**Authors:** Olufunmilola Olubisi Abolurin, Tinuade Adetutu Ogunlesi, Emmanuel Damilare Ajibola, Adesola Olubunmi Adekoya, Olutomiwa Ayoola Omokore, Fisayo Grace Ajayi, Collins Chijioke Adumah

**Affiliations:** 1 Department of Pediatrics, Babcock University Teaching Hospital, Ilishan, Ogun State, Nigeria,; 2 Department of Pediatrics, Olabisi Onabanjo University, Sagamu, Ogun State, Nigeria,; 3 Department of Family Medicine, Babcock University Teaching Hospital, Ilishan, Ogun State, Nigeria,; 4 Department of Pediatric Surgery, Obafemi Awolowo University, Ile-Ife, Osun State, Nigeria

**Keywords:** Morbidity, mortality, neonate

## Abstract

**Introduction:**

neonatal mortality in Nigeria is still high and the primary causes are largely preventable. The study was carried out to assess the pattern of morbidity and mortality among hospitalized Nigerian neonates.

**Methods:**

a retrospective study was carried out on neonates admitted into the Special Care Baby Unit of the Babcock University Teaching Hospital, Ilishan-Remo, over five years between January 2014 and December 2018. The patients´ case files were retrieved from the health records unit and relevant information on their morbidities and outcomes were extracted. Bivariate analyses and logistic regression analyses were done to determine factors associated with mortality.

**Results:**

a total of 490 babies were studied, of which 276 (56.3%) were males. Nearly two-thirds of the babies were inborn (309; 63.1%), 344 (70.2%) were delivered at term, and 136 (27.8%) were low birth weight babies. The median gestational age at birth was 37 weeks. Perinatal asphyxia was the most common diagnosis necessitating admission (104; 21.2%), closely followed by neonatal jaundice. Sepsis and prematurity were also common diagnoses. Mortality occurred in 39 (8.0%) babies; the highest case fatality rates were recorded among preterm babies (13/70; 18.6%) and those who had perinatal asphyxia (16/104; 15.4%). On logistic regression analysis, outborn status (aOR= 2.51, 95% CI= 1.26 - 5.01, p= 0.009) and preterm delivery (aOR= 4.10, 95% CI= 2.06 - 8.16, p<0.001) had significant independent association with mortality.

**Conclusion:**

perinatal asphyxia and prematurity were the leading causes of death among the neonates studied. Efforts to reduce neonatal mortality by preventing these morbidities and promptly initiating appropriate treatment, when present, should be heightened to reduce neonatal mortality.

## Introduction

The neonatal period is a highly vulnerable period that heralds the beginning of extra-uterine life and involves several physiological adjustments. It is associated with higher morbidity and mortality, much more than any other childhood period [1]. Annually, about 2.5 million neonates die worldwide, accounting for nearly half of all deaths among children under five years of age [2]. Hence, the pattern of morbidity and mortality in the newborn period contributes significantly to the under-five survival indices [2]. This reflects how important neonatal morbidity and mortality reduction is to the overall reduction of childhood indices. In Nigeria and other developing countries, the situation is more severe as neonatal mortality is way higher and the primary causes are largely preventable [2]. Generally, global neonatal mortality rates have declined substantially since the year 2000; however, the deaths are densely concentrated in South Asia and sub-Saharan Africa, where over half of the global neonatal mortalities occur [2,3]. In these countries, about one in every 36 newborns is likely to die within the first month of life as against one in 333 in developed, high-income societies. Unfortunately, Nigeria is reportedly one of the countries that have the highest neonatal mortality rates worldwide [3]. For the 2030 timeline target of the sustainable development goal (SDG) for under-five mortality to be met, the countries where the burden is highest, including Nigeria, must target improved child health indices [3]. This can only be possible if the causes of neonatal mortality, which are responsible for the bulk of under-five deaths, are adequately addressed [3].

Evidence has shown that with improved neonatal care, morbidity and mortality in this period can be greatly reduced. This includes prompt identification and appropriate management of the medical problems occurring in the newborn period [2,3]. Neonatal morbidity patterns vary widely, even across similar geographical climes [2,3]. Similarly, the rates and causes of neonatal mortality also vary worldwide and across different communities, states and geopolitical zones, even within Nigeria [4-11]. The patterns are influenced by medical, socio-demographic, cultural, and environmental factors [12], hence, the need to document the local morbidity patterns. The leading causes of neonatal deaths in the United States and other developed, high-income societies are severe congenital malformations and preterm birth complications [2]. In contrast, most cases in our environment are from asphyxia, prematurity, neonatal jaundice, and infections such as sepsis and tetanus [5-8]. It has been quite a challenge dealing with these aetiologies despite being largely preventable and/or readily treatable by cost-effective methods [3]. Most of the available data on neonatal morbidity and mortality in Nigeria are non-recent and may not be reflective of the recent trends. Identifying current patterns can inform institutional, local, and regional planning of interventions towards achieving a reduction in neonatal mortality, and possibly serve as a tool for national advocacy as Nigeria strives to further reduce the scourge of neonatal and childhood mortality. This may help to improve the country´s health indices with a focus on the Sustainable Development Goals (SDGs). This study aimed to assess the pattern of morbidity and mortality among neonates admitted into the Special Care Baby Unit (SCBU) of the Babcock University Teaching Hospital (BUTH) over five years from January 2014 to December 2018.

## Methods

**Study design:** the study was a retrospective study that was carried out on neonates admitted into the SCBU of the Babcock University Teaching Hospital (BUTH), Ilishan-Remo, Ogun state, Nigeria over five years. Both inborn (babies delivered within our facility) and outborn babies (those delivered outside our hospital) were included.

**Study setting:** the study involved neonates admitted into BUTH over five years spanning between January 2014 and December 2018. The hospital is a private tertiary healthcare institution owned by Babcock University, a Seventh-day adventist institution of higher learning. It is located in the Ikenne Local Government Area of Ogun state and serves the host community as well as neighboring towns and states. The Special Care Baby Unit (SCBU) of the hospital provides 24-hour neonatal care services. It is run by two consultant neonatologists alongside resident doctors. It also includes nursing staff that have been trained in pediatric/neonatal care. It has both inborn and outborn sections with a total of 10 cots and 10 incubators. The SCBU of BUTH was established in the year 2014.

**Study population:** the study population included all neonates admitted into the SCBU of BUTH during the study period. The inclusion criteria were babies admitted within the first 28 days of life, both inborn and outborn. Neonates brought in dead and those in transit to other tertiary centres were excluded.

**Data collection:** the patients´ case files were retrieved from the health records unit and relevant information, including age at admission, gender, gestational age at birth, birth weight, place of birth, diagnosis at admission, duration of admission, associated problems during admission, and outcome of admission, were extracted. These were filled into a proforma and subsequently entered into a data analysis software program.

**Definitions:** the neonates were classified as preterm (<34 weeks), near-term (34-36 completed weeks), term (37-41 completed weeks) or post-term (≥42 weeks) based on their gestational age at delivery [13]. Those who had birthweight less than 2500g were categorized as low birth weight (LBW) and further sub-classified as very low birth weight (VLBW) when birthweight was between 1000g and 1499g or as extremely low birth weight (ELBW) when less than 1000g [14].

**Statistical analysis:** data was analyzed using the Statistical Package for the Social Sciences (SPSS) software version 22.0. Neonates whose hospital records could not be accessed and those with incomplete data were excluded from the analysis. Descriptive statistics were used to represent and describe variables using tables and charts. Student t-test and Pearson´s Chi-square (χ^2^) test were used to compare continuous and categorical variables, respectively. Logistic regression analysis was done to determine factors independently associated with mortality; only factors that were statistically significant on bivariate analysis were included in the logistic regression model. The level of statistical significance was set at p <0.05.

**Ethical considerations:** ethical clearance for the study was obtained from the Babcock University Health Research Ethics Committee (BUHREC 566/19). The identity of each participant was kept confidential and all information obtained was assigned code numbers, pass-worded, and kept secret.

## Results

**General characteristics of the study population:** a total of 852 babies were admitted into the SCBU over the five years under review. Adequate records for analysis were available for 516 of these babies, necessitating the exclusion of the 336 babies with missing/incomplete data. Twenty-six babies were further excluded because they were admitted only for brief periods of observation after cesarean section and had no definite medical problem. Hence, 490 babies were included in the study. In Table 1 shows the characteristics of the 490 babies. Nearly two-thirds were inborn (309; 63.1%). Private hospitals featured as the most common place of birth among the outborn (72; 14.7%). More than half (276; 56.3%) of the babies were males, and cesarean deliveries were common among them (52.9%). Three hundred and forty-four (70.2%) of them were delivered at term, 22 (4.5%) were macrosomic, 136 (27.8%) were low birth weight (LBW) babies, of which 26 were very low birth weight (VLBW) and 13 were extremely low birth weight (ELBW). Three hundred and twelve (63.7%) of the babies were admitted within the first 24 hours of life. Among those admitted after 24 hours of life, age at admission ranged from 1 to 28 days with a mean of 4.5 ± 4.6 days. The age at admission was significantly higher among the outborn babies, with only 42.0% of them admitted within the first 24 hours, compared with 76.4% of the inborn babies (p <0.001).

**Table 1:** characteristics of the 490 babies studied

Characteristic	Number (%)
**Source of admission**	
Inborn	309 (63.1)
Outborn	181 (36.9)
**Place of birth**	
BUTH	309 (63.1)
Private hospital	72 (14.7)
General hospital	23 (4.7)
Maternity home	18 (3.7)
Primary health care	16 (3.3)
Home	9 (1.8)
Government-owned tertiary hospital	5 (1.0)
Traditional birth attendant’s centre	5 (1.0)
Not available	33 (6.7)
**Mode of delivery**	
Spontaneous vaginal delivery	199(40.6)
Caesarian section	259 (52.9)
Vacuum-assisted delivery	5 (1.0)
Forceps-assisted delivery	5 (1.0)
Assisted breech delivery	5 (1.0)
**Sex**	
Male	276(56.3)
Female	214 (43.7)
**Duration of gestation**	
Term	344(70.2)
Preterm	65 (13.3)
Late preterm	75 (15.3)
Post term	6 (1.2)
**Birth weight category**	
Normal	315(64.3)
Low birth weight	136 (27.8)
Macrosomic	22 (4.5)
Missing	17 (3.5)
**Age at admission**	
<24 hours	312 (63.7)
1-7 days	150 (30.6)
8-24 days	28 (5.7)

BUTH: Babcock University Teaching Hospital

Perinatal asphyxia was the most common diagnosis necessitating admission (104; 21.2%), closely followed by neonatal jaundice (NNJ) (98; 20.0%) as shown in Table 2. Sepsis, respiratory distress (mostly due to transient tachypnoea of the newborn and aspiration), and prematurity were also common diagnoses. Others included ophthalmia neonatorum, macrosomia, omphalocele, omphalitis, mastitis, dehydration fever, and vomiting precipitated by swallowed maternal products. Furthermore, several of the babies who were admitted for other reasons besides NNJ developed varying degrees of jaundice during admission and were treated accordingly. Perinatal asphyxia and prematurity were relatively more common diagnoses among the outborn babies, whereas jaundice, respiratory distress, and hypoglycemia were more common among the inborn babies as shown in Table 2. Ninety-three (19.0%) of the 490 babies received blood transfusion during admission, of which 40 (8.2%) had exchange blood transfusion (EBT). The majority (411; 83.9%) of the babies were discharged home, 30 (6.1%) got discharged against medical advice (DAMA), 10 (2.0%) were referred to other tertiary health facilities for further care, and 39 (8.0%) died. The duration of admission ranged from 1 to 58 days with a mean of 9.5 ± 10.5 days; it was <7 days in 282 (57.6%), 7 - 14 days in 123 (25.1%) and >14 days in 85 (17.3%) babies. Only about one-tenth (10.9%) of the babies who survived attended the follow-up clinic. Eighteen (46.2%) of the 39 deaths occurred within 24 hours of admission, while 7 occurred after 24 hours, but within 48 hours of admission; 8 (20.5%) occurred >7 days after admission. Annual mortality rate ranged between 5.2% - 10.0%, but there was no statistically significant difference in the mortality rates across the five years of the study (p = 0.694). Perinatal asphyxia (41.0%) and prematurity (33.0%) jointly accounted for nearly three-quarters of the mortalities recorded.

**Table 2:** morbidity pattern among the babies admitted into the neonatal unit

Diagnosis/indication for admission	Inborn (309) n (%)	Outborn (181) n (%)	Total (490) n (%)
Perinatal asphyxia	56 (18.1)	48 (26.5)	104 (21.2)
Neonatal jaundice	67 (21.7)	31 (17.1)	98 (20.0)
Neonatal sepsis	49 (15.9)	29 (16.0)	78 (15.9)
Respiratory distress	62 (20.1)	13 (7.2)	75 (15.3)
Prematurity	31 (10.0)	39 (21.5)	70 (14.3)
Neonatal hypoglycaemia	26 (8.4)	3 (1.7)	29 (5.9)
Neonatal seizures	1 (0.3)	4 (2.2)	5 (1.0)
Others	17 (5.5)	14 (7.7)	31 (6.3)

χ^2^ = 41.63, p <0.001

**Mortality and associated factors:** Table 3 shows the case fatality rates (CFR) in relation to diagnosis, place of birth, gestational age, and birth weight. The highest CFR was recorded among preterm babies (18.6%) and those who suffered from perinatal asphyxia (15.4%) (p<0.001). Mortality was significantly higher among the outborn babies, compared with the inborn (13.3% vs 4.9%; p= 0.001). Twenty-four (61.5%) of the 39 babies who died were LBW, of which 12 were ELBW; lower birth weight was significantly associated with an outcome of death (p <0.001). Mortality was also significantly higher among the preterm and the late preterm babies, compared with those who were term (p <0.001). On logistic regression analysis, outborn status and preterm delivery had significant independent association with mortality; outborn babies were two times more likely to die than inborn babies (aOR= 2.51, 95% CI = 1.26 - 5.01, p= 0.009), and preterm babies four times more likely to die than term babies (aOR= 4.10, 95% CI= 2.06 - 8.16, p <0.001) as shown in Table 4.

**Table 3:** case fatality rates in relation to diagnosis, source of admission, gestational age, and birth weight

	Outcome	
	Died n (%) *	Survived n (%)
**Diagnosis**		
Prematurity	13 (18.6)	57 (81.4)
Perinatal asphyxia	16 (15.4)	88 (84.6)
Sepsis	6 (7.7)	72 (92.3)
Neonatal jaundice	2 (2.0)	96 (98.0)
Respiratory distress	0 (0.0)	75 (100.0)
	χ2 = 32.80, p <0.001	
**Source of admission**		
Inborn	15 (4.9)	294 (95.1)
Outborn	24 (13.3)	157 (86.7)
	χ2 = 11.01, p = 0.001	
**Duration of Gestation**		
Preterm	16 (24.6)	49 (75.4)
Late preterm	8 (10.7)	67 (89.3)
Term	15 (4.4)	329 (95.6)
Post-term	0 (0.0)	6 (100.0)
	χ2 = 31.97, p <0.001	
**Weight category**		
ELBW	12 (92.3)	1 (7.7)
VLBW	1 (3.8)	25 (96.2)
LBW	11 (11.3)	86 (88.7)
Normal weight	10 (3.2)	305 (96.8)
Macrosomic	0 (0.0)	22 (100.0)
	χ2 = 153.43, p <0.001	


* Case fatality rate is equivalent to the percentage of babies who died in each category; ELBW: extremely low birth weight; VLBW: very low birth weight; LBW: low birth weight

**Table 4:** logistic regression analysis for factors independently associated with mortality

Variable		aOR (95% CI)	P-value
Source of admission	Outborn; inborn (reference)	2.51 (1.26 - 5.01)	0.009
Preterm birth	Preterm; not preterm (reference)	4.10 (2.06 - 8.16)	<0.001

aOR: adjusted odds ratio; CI: confidence intervals

## Discussion

This study sought to assess the pattern of morbidity and mortality among neonates in a private tertiary health facility. Perinatal asphyxia was the most common diagnosis necessitating admission, followed by neonatal jaundice. Mortality occurred in nearly one-tenth of the babies, with the highest case fatality rates observed among preterm babies and those who suffered perinatal asphyxia. Mortality was significantly higher among the outborn and preterm babies. A total of 852 babies were admitted into the newborn unit over five years in this study. The average annual admission rate was relatively small compared to previous studies conducted in government-owned hospitals [4-7]; it was however similar to that observed in a private health facility [8]. The higher cost of care in private health facilities may limit the number of patients presenting for care, considering the country's high poverty level. Furthermore, our study covered the first few years following the inception of the hospital, thus the level of awareness of its existence might have been low at the time. We found a predominance of inborn babies who constituted nearly two-thirds of all the babies admitted during the study period. This is similar to the neonatal admission pattern observed in a government-owned teaching hospital in Benin City [7], whereas, other centers have mostly reported nearly equal admission rates of inborn and outborn babies [5,9,10]. The presence of other health facilities that provide specialized neonatal care in close proximity to our hospital may explain the predominance of inborn babies; which also applies to the Benin hospital with a similar inborn admission pattern [7]. The age at admission was also generally lower among the inborn babies, as observed in a previous study [10]. This finding may be attributable to the availability of pediatric specialists at the study centers, which would aid the early identification of neonatal problems that may portend danger, resulting in prompt admission of such babies. On the other hand, non-pediatrician health workers practicing in non-specialist health facilities may miss some early danger signs in neonates and may not refer such babies until more serious signs appear.

We found perinatal asphyxia as the most common diagnosis necessitating admission, closely followed by neonatal jaundice; Sepsis, prematurity, and respiratory distress were also common morbidities. This is in keeping with the findings in previous Nigerian studies, which all reported similar morbidity patterns among admitted newborns [4-10]. However, neonatal jaundice was relatively more frequent as the primary diagnosis in our study, whereas it featured mostly as the third or fourth morbidity, after asphyxia, sepsis, and sometimes prematurity, in many of the previous studies [4,6,7,10]. Furthermore, babies who had respiratory distress constituted a significant proportion of the babies we studied, despite excluding those whose dyspnoea was attributable to sepsis, asphyxia or respiratory distress syndrome (prematurity-related) from this category. This was not a common pattern in previous studies and may be partly explained by the fact that transient tachypnoea of the newborn, meconium aspiration, and aspiration of maternal products such as liquor or blood, were categorized together in the present study. The morbidity pattern varied significantly between the inborn and outborn babies, with perinatal asphyxia and prematurity taking the lead among the outborn, as opposed to jaundice and respiratory distress among the inborn. The leading problems among inborn babies are conditions that are likely to be deliberately looked out for by pediatricians during routine reviews in the early neonatal period but may be overlooked or ignored by healthcare providers in non-specialist hospitals, who may not raise concerns until there is worsening severity of such features. It is well known that neonatal jaundice is most often physiologic, in which case self-resolution is common [15].

On the other hand, sepsis, which might have been expected to be more prevalent among the outborn babies, featured almost equally among the inborn and outborn. This may be explained by the fact that babies who had a diagnosis of presumed sepsis (presence of maternal risk factors such as prolonged rupture of membranes, but no clinical features), which was likely more common among the inborn, were not classified separately from those who had clinical features of sepsis. Furthermore, the higher prevalence of hypoglycemia among the inborn babies was likely related to the practice of routine blood glucose checks for neonates in the first few days of life. Neonatal tetanus featured as a common neonatal morbidity in some earlier studies [6-9] but was not recorded in the present study. Similarly, more recent studies did not report tetanus as one of the morbidities observed among admitted newborn babies [5,10,11]. This may mean that its prevalence is declining, which would be favorable for the proposed elimination of the disease. The coverage of maternal tetanus toxoid injections might have improved over the last decade before which the earlier studies were conducted. On the other hand, it is also possible that babies with tetanus, who are more likely to be from socio-economically deprived backgrounds, would rather present at public health institutions rather than at private ones which are generally more expensive. Nearly one-fifth of the babies studied had blood transfusions, with almost half of the transfusions being EBT. The transfusion rate observed was lower than the rate reported among admitted neonates in Sagamu (27.9%) [16], which is a neighboring town, and also in Zaria (34.9%) [17], Northwest Nigeria. A much lower neonatal transfusion rate of 11.7% was however reported in Ogbomoso [18], Oyo State, Nigeria.

The differences may be related to the varying distribution of morbidities among the neonates studied, especially in relation to the prevalence of jaundice, prematurity, and sepsis, which are frequently associated with anemia and often necessitate blood transfusion in neonates [16-18]. Exchange blood transfusion accounted for more than half of the transfusions in two of the earlier studies [16,18], whereas about one-third of the transfused babies in the Zaria study had EBT [17]. Again, the disparity may be reflective of the morbidity distribution in the study population, bearing in mind that severe neonatal jaundice (NNJ) is the most common indication for neonatal EBT [19]. The rate of DAMA in our study (6.1%) was similar to those reported in some previous studies [6,11], but lower than in others (12.4 - 15%) [5,8], while some other studies found much lower DAMA rates ranging between 1.7 - 4.0% [4,7,9]. Most DAMA decisions are usually due to financial constraints, though sociocultural factors are also frequently involved [20]. Considering the relatively higher cost of healthcare in the private setting, a higher rate of DAMA would have been expected in the present study, compared with studies conducted in government-owned facilities; this was however not a striking observation, suggesting that the cost of the services was likely affordable. Perinatal asphyxia and prematurity were the leading causes of death among the neonates we studied. Furthermore, prematurity and being outborn were independent predictors of mortality. These findings are in agreement with previous studies which revealed that asphyxia and prematurity, along with sepsis, were the three leading causes of neonatal deaths [4-11].

These conditions are largely preventable, and death from them can also be avoided when they are promptly and appropriately managed [2,3]. Among the outborn babies, late presentation was a contributory factor. The neonatal mortality rate in the present study was 8.0%, which is comparable to the 6 - 7% reported by Onazi *et al*. [4], and Abdullahi *et al*. [11]. However, the mortality rates found in most other previous studies were much higher, ranging from 12.4% to 20.3% [5-10]. Our facility, being privately owned, has access to better funding, compared to many public facilities. Hence, the capability of attracting better facilities, equipment, and expertise is higher, such that supportive investigations, particularly imaging studies, are more readily available. This might have increased the accuracy of the final diagnosis and set the template for better management, possibly resulting in better survival rates among babies managed in our facility. On the other hand, the varying distribution of morbidities in the different studies may also account for this disparity, as morbidities such as asphyxia and prematurity may carry higher mortality rates, especially when the babies are VLBW or ELBW [2,6-8]. Therefore, the importance of prompt referral of babies with these problems to pediatric specialists cannot be over-emphasized. In addition, overall improvement in neonatal care, especially during birth and its immediate period, will go a long way in reducing neonatal morbidity and mortality in the country, paving the way for achieving SDG goal 3 [3]. This study is limited by its retrospective nature, as incomplete data necessitated the exclusion of some of the babies admitted during the study period.

## Conclusion

Perinatal asphyxia and prematurity were the leading causes of death among the neonates studied and prematurity had a significant independent association with mortality on logistic regression. Efforts towards preventing these morbidities and promptly initiating appropriate treatment should be heightened to reduce neonatal mortality.

### What is known about this topic

- The neonatal mortality rate is high in Nigeria;
- Asphyxia, prematurity, and infections are common causes of morbidity and mortality among Nigerian neonates.

### What this study adds

- Neonatal morbidity and mortality patterns in Nigeria have remained essentially the same over time, with asphyxia and prematurity being the leading causes;
- Tetanus may no longer be a common cause of neonatal morbidity and mortality in Nigeria.
